# Improving the Energetic Stability and Electrocatalytic Performance of Au/WSSe Single-Atom Catalyst with Tensile Strain

**DOI:** 10.3390/nano12162793

**Published:** 2022-08-15

**Authors:** Shutao Zhao, Xiao Tang, Jingli Li, Jing Zhang, Di Yuan, Dongwei Ma, Lin Ju

**Affiliations:** 1 Key Laboratory of Functional Materials and Devices for Informatics of Anhui Higher Education Institutes, School of Physics and Electronic Science, Fuyang Normal University, Fuyang 236037, China; 2 College of Science, Institute of Materials Physics and Chemistry, Nanjing Forestry University, Nanjing 210037, China; 3 School of Physics and Electrical Engineering, Anyang Normal University, Anyang 455000, China; 4 Key Laboratory for Special Functional Materials of Ministry of Education, School of Materials Science and Engineering, Henan University, Kaifeng 475004, China

**Keywords:** single-atom catalyst, Au/WSSe, electrocatalysis, tensile strain

## Abstract

In the areas of catalysis and renewable energy conversion, the development of active and stable electrocatalysts continues to be a highly desirable and crucial aim. Single-atom catalysts (SACs) provide isolated active sites, high selectivity, and ease of separation from reaction systems, becoming a rapidly evolving research field. Unfortunately, the real roles and key factors of the supports that govern the catalytic properties of SACs remain uncertain. Herein, by means of the density functional theory calculations, in the Au/WSSe SAC, built by filling the single Au atom at the S vacancy site in WSSe monolayer, we find that the powerful binding between the single Au atom and the support is induced by the Au *d* and W *d* orbital hybridization, which is caused by the electron transfer between them. The extra tensile strain could further stabilize the Au/WSSe by raising the transfer electron and enhancing the orbital hybridization. Moreover, by dint of regulating the antibonding strength between the single Au atom and H atom, the extra tensile strain is capable of changing the electric-catalytic hydrogen evolution reaction (HER) performance of Au/WSSe as well. Remarkably, under the 1% tensile strain, the reaction barrier (0.06 eV) is only one third of that of free state. This theoretical work not only reveals the bonding between atomic sites and supports, but also opens an avenue to improve the electric-catalytic performance of SACs by adjusting the bonding with outer factors.

## 1. Introduction

As a new family of catalysts, single-atom catalysts (SACs) typically offer isolated active sites, great selectivity, and simplicity in separation from reaction systems. They have recently sparked a lot of attention throughout the globe due to their distinctive structural characteristics, which include optimized metal utilization, customized active sites, and astonishing catalytic activities [1,2,3,4,5,6]. Nevertheless, due to their high surface energy, single-atom sites are prone to sintering and aggregating into thermodynamically stable nanoclusters [7,8]. Sintering must be averted by adding the proper supports to enhance the local coordination environment, electrical characteristics, and strong metal-support interactions.

The recent emergence of Janus two-dimensional (2D) transition metal dichalcogenides (TMDs), which refer to layers with different surfaces (e.g., MoSSe and WSSe), have piqued intense research interest due to their distinctive characteristics and potential energy conversion applications [9,10,11,12]. The intrinsic dipole in Janus 2D materials induced by the out-of-plane asymmetric structure could strengthen the adsorption of molecules or atoms on the surface [13], which might result in a better production environment for SACs [14]. Transition-metal adatoms, in turn, could efficiently adjust the Janus TMDs’ dipole moments [15]. It has been claimed that, by increasing the inherent dipole, the adsorption of appropriate transition-metal adatoms can cause the interactions between H_2_O and MoSSe to transform from weak electrostatic van der Waals (vdW) to powerful chemical bonding, considerably enhancing the adsorption of H_2_O molecules and laying the groundwork for photocatalytic water-splitting processes [14].

Additionally, although a range of potential SACs with inexpensive supports are emerging as appealing candidates for heterogeneous catalysis [16,17], it is unclear which precise functionalities and important roles of the supports really impact the catalytic capacities of these SACs. For the purpose of investigating the bonding between atomic sites and supports and the effect of bonding on improving the catalytic performance, in this work, we chose single Au atom and Janus WSSe monolayer to construct SAC, and study the interaction between them by analyzing interfacial transfer electron and electronic orbital coupling. Moreover, we applied external tensile strain to further stabilize the SAC and adjust the electric-catalytic performance for hydrogen evolution reaction (HER).

## 2. Calculation Method

In this study, we performed the DFT calculations for both geometrical relaxations and electronic structures using the Vienna Ab initio Simulation Package (VASP) program (Version 5.3, Hanger Group, University of Vienna) [18,19]. To represent the electron–ion interaction, we employed the projector augmented wave (PAW) pseudo potentials [20,21]. As the exchange-correlation functional, we selected generalized gradient approximations of Perdew–Burke–Ernzerhof (GGA-PBE) [22]. We placed a 20 Å vertical vacuum interval between each sample and the nearby mirror images to prevent interactions. We used Grimme’s DFT-D3 method to deal with the vdW force [12,23]. A 3 × 3 × 1 gamma-pack k-mesh regulated the Brillouin zone. The convergence conditions for the force and energy were 10^−2^ eV/Å and 10^−5^ eV, respectively, with the cutoff energy set at 500 eV. Despite the fact that tungsten is a heavy metal, since the influence of spin-orbital coupling (SOC) on the band gap of the WSSe monolayer has been shown to be minimal [12], we did not use the SOC correction in this instance to conserve computational resources. Additionally, the computational hydrogen electrode (CHE) model was used to perform the Gibbs free energy calculation [24]; meanwhile, the implicit solvent model included in VASPsol was used to account for the solvent effect [25,26]. In the Supporting Information, more Gibbs free energy simulation specifics (as listed in Table S1) are provided.

## 3. Results and Discussion

Introducing Au single-atom or cluster into electro- and photo-catalysts by doping or adsorbing has been demonstrated as an efficient approach to improve the catalytic performance. For the electrocatalysis, the accession of Au not only increases the conductivity of the catalyst, but also causes a strong electronic interaction at their interface, boosting the electrocatalytic performance of many compounds, such as Au/Ni_3_S_2_ [27] and Au/TiO_2_ [28,29]. As to the photocatalysis, due to the prevention of charge recombination in the area of the space charge, together with the prolonged light absorption caused by the surface plasmon resonance (SPR) effect, loading Au could greatly improve visible light catalytic activity in many systems, such as 2% Au loaded SnO_2_/g-C_3_N_4_ [30] and Au decorated WO_3_/g-C_3_N_4_ [31].

In our work, for the SAC constructed by adding single Au atom on WSSe monolayer, generally, there may be two main configurations. Specifically, one is that the single Au atom adsorbs on the surface of WSSe monolayer (case 1); the other is that the single Au atom replaces one of the atoms on the surface of WSSe monolayer (case 2). Hence, first of all, we confirmed the more favorable configuration by comparing the adsorption energy (*E*_ad_) of case 1 with the formation energy (*E*_for_) of case 2, respectively. Lower value indicates more stable. After that, we studied the interaction between them through analyzing interfacial transfer electron and electronic orbital coupling. Moreover, we applied external tensile strain with the purpose of further stabilizing the SAC and tuning the electric-catalytic HER performance.

### 3.1. Single Au Atom Adsorbed Janus WSSe Monolayer

**Figure 1:**
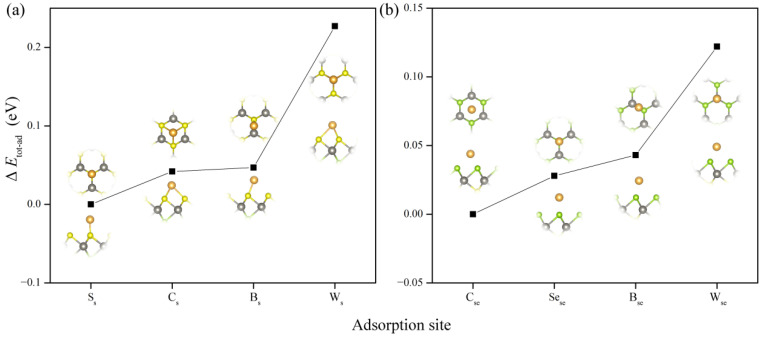
Relative total energy (Δ*E*) of single Au atom adsorbed WSSe monolayer with the four adsorption sites on the (**a**) S and (**b**) Se layers, respectively. For each case, the energy of the system with the lowest total energy is taken as the reference value. The insets show the top view (above) and the side view (below) of the optimized structures of these systems. The yellow, green, grey, and golden balls represent S, Se, W and Au atoms, respectively.

To pursue the energetically stable configuration of SAC built by single Au atom adsorbed Janus WSSe monolayer, as shown in Figure S1, we took two absorption cases into consideration, namely the single Au atom adsorbing on the Se and S sides, respectively. For each absorption case, we examined four possible adsorption sites, namely centre, bond, S (Se), and W sites labeled as C_s_, B_s_, S_s_ and W_s_ for S layer, and C_se_, B_se_, Se_se_ and W_se_ for Se layer, respectively. The optimized structures for all these samples are shown in the inset of Figure 1. Based on the total energy of these systems, we found that, on the S layer, the single Au atom tended to stay on the S_s_ site; meanwhile on the Se layer, it was likely to locate at the C_se_ site. Furthermore, we computed the *E*_ad_ to estimate the adsorption strength, which is defined as follows,

(1) $$E_{\mathrm{ad}}=E_{\mathrm{ad}\_\mathrm{sys}}-E_{\mathrm{sub}}-E_{\mathrm{Au}}$$

where $E_{\mathrm{ad}\_\mathrm{sys}}$ and $E_{\mathrm{sub}}$ are the total energy of WSSe monolayer with and without single Au atom adsorption. $E_{\mathrm{Au}}$ equals to −3.274 eV, which is the total energy of per Au atom in stable Au solid, obtained from the Materials Project database. The *E*_ad_ of single Au atom adsorbed on Ss and Cse sites are 0.763 eV and 0.834 eV. On the basis of the definition of $E_{\mathrm{ad}}$, the smaller value demonstrates a higher stability. That is to say, the single Au atom prefers to stay at the S layer, which may be explained by the higher electronegativity of S element (5.85 eV) than the one of Se element (5.76 eV) [32]. As shown in Figure 1a, the single Au atom could be fixed by its near S atoms through chemical bonds. However, the positive values of *E*_ad_ indicate that all these adsorption models are not stable. The single Au atom adsorbed on the surface of WSSe probably aggregates into cluster, which means that single Au atom adsorption is not a feasible way to make up SAC.

### 3.2. Single Au Atom Doped Janus WSSe Monolayer (Au/WSSe)

In the WSSe monolayer, there are three kinds of atoms, namely W, S, and Se atoms, which means that the single Au atom has three choices to replace one atom of the WSSe monolayer. Before this substitution process, a W/S/Se vacancy should first be formed [33]. According to our previous work [34], the formation energy of S vacancy (0.49 eV/atom) is much lower than the ones of Se (3.77 eV/atom) and W (4.97 eV/atom) vacancies. Therefore, herein, we only consider the case that the single Au atom fills in the S vacancy. The $E_{\mathrm{for}}$ for this situation is gained by the following equation,

(2) $$E_{\mathrm{for}}=E_{\mathrm{doped}\_\mathrm{sys}}-E_{\mathrm{vac}-s}-E_{\mathrm{Au}}$$

where $E_{\mathrm{doped}\_\mathrm{sys}}$ and $E_{\mathrm{vac}-s}$ are the total energy of Au/WSSe and WSSe monolayer with one S vacancy, respectively. The calculated $E_{\mathrm{for}}$ is −0.55 eV. This negative value means the S vacancy could work as an anchored site to capture a single Au atom, which is similar to the case of Pt single atoms on the nanosized onion-like carbon (Pt_1_/OLC) [35]. Moreover, by running ab initio molecular dynamics simulations (AIMD) with the Nos’e thermostat model as implemented in VASP, the thermal stability of Au/WSSe was evaluated. The outcome of the AIMD simulations on Au/WSSe is depicted in Figure S2, where the negligible geometric reconstructions and small total energy variations suggest that Au/WSSe may be thermostable at ambient temperature. Besides the Au element, we also considered the single atom of its congeners (i.e., Ag and Cu) to build SACs. However, as shown in Figure S3, both their formation energies were positive, indicating they are not as stable as Au/WSSe. Therefore, we do not discuss the cases of Ag/WSSe and Cu/WSSe further.

**Figure 2:**
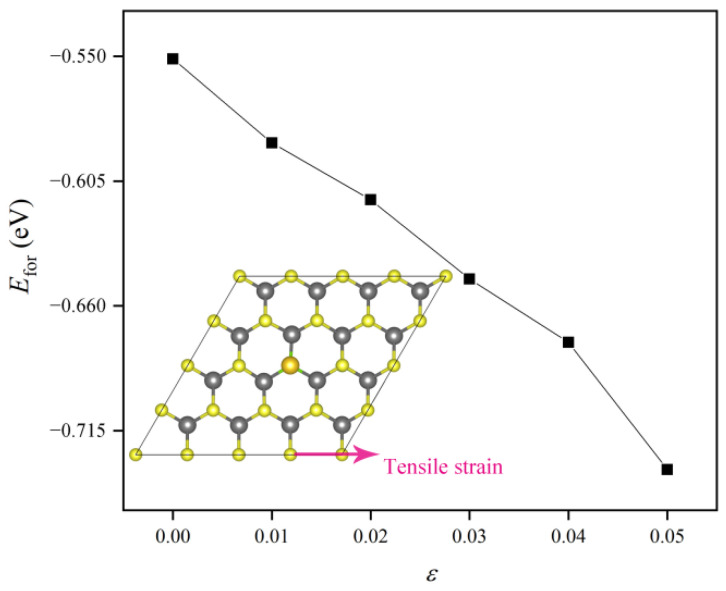
Formation energy of Au/WSSe under different tensile strain.

**Figure 3:**
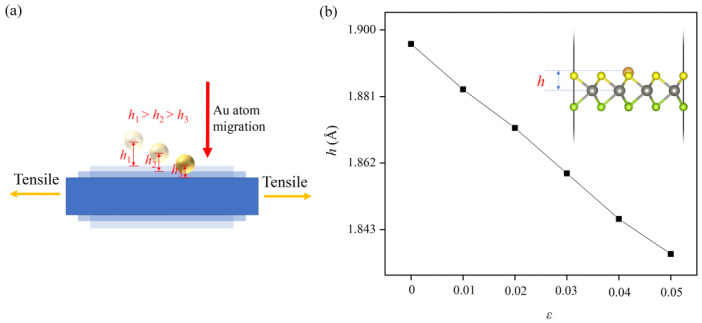
(**a**) The diagram of single Au atom position change over external tensile stress. (**b**) The height of the single Au atom from the W plane in Au/WSSe under different tensile strain.

To further enhance the stability of Au/WSSe, we applied uniaxial tensile strain to it, which is widely used to tune the morphologic and electric structures of two dimensional materials [10,12,36,37]. As displayed in Figure 2, the calculated results stated that the *E*_for_ became lower with higher tensile strain. Moreover, as shown in Figure 3a, the single Au atom came closer to the surface of the WSSe monolayer as the tensile strain rose, which could be verified by the lower height (*h*) of the single Au atom from the W plane (see Figure 3b). Furthermore, based on the calculated results obtained with the climbing image nudged elastic band (CI-NEB) method (see Figure S4), we found that the extra tensile strain could make the diffusion barriers of single Au atom increase (from 2.75 eV at free state to 3.10 eV under 5% tensile strain), which also revealed that introducing uniaxial tensile strain could increase the stability of Au/WSSe.

### 3.3. Interfacial Interaction between Single Au Atom and WSSe Monolayer

As plotted in Figure S5, compared with the case of the pristine WSSe monolayer, the results of total density of states (TDOS) demonstrated that both the adsorption and dopant of a single Au atom could induce obvious spin-splits in the gap states at/near the Fermi level, which caused magnetic moments of 1.00 and 0.94 µB, respectively. As mentioned earlier, the doped system is more stable than the adsorption one; therefore, we focused on the Au/WSSe and explored the origin of its tensile strain-dependent stability by researching the interfacial interaction between the single Au atom and WSSe monolayer, which could be reflected from the electronic orbitals coupling. As plotted in Figure S5c, the TDOS shows that there was one obvious orbital hybridization peak near the Fermi level in each spin direction. In addition, we assessed the accurate electronic structure of Au/WSSe using the Heyd–Scuseria–Ernzerhof (HSE06) hybrid functional [38] to prevent the underestimation of the band gap calculated within PBE. As shown in Figure S6, the gap state near the Fermi level was dominated by the spin-up states, which agreed with the PBE result.

**Figure 4:**
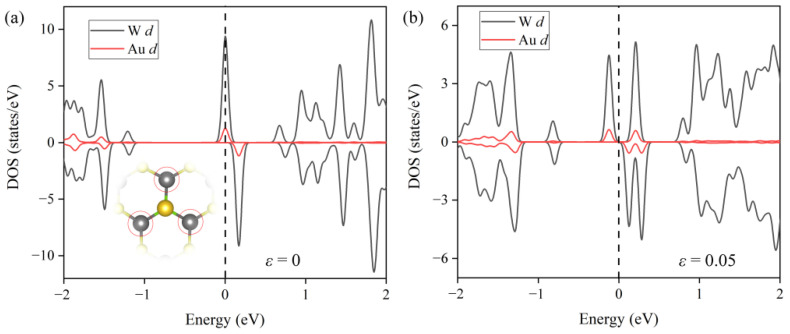
Partial density of states (PDOS) of the *d* orbitals of the single Au atom and its three nearest neighboring W atoms (circled with the red lines shown in the inset) in Au/WSSe (**a**) without and (**b**) with external tensile strain (ε = 5%).

Based on the analysis of PDOS, we found that this orbital hybridization was mainly contributed by the coupling of the *d* orbitals of the single Au atom and its three nearest neighboring W atoms (see Figure 4a). Numerous additional SACs have also been identified to exhibit the strong contact between atomic species and the nearby atoms on the support [39,40,41]. Notably, when the external tensile strain was (ε = 5%) applied, as shown in Figure 4b, the orbital hybridization peak near the Fermi level split into two peaks, broadening the coupling energy range, which surely strengthened the interfacial interaction between the single Au atom and WSSe monolayer. Furthermore, the expansion of gap-states indicates an enhanced electric conductivity, which could be confirmed by experimentally measuring the I-V curve.

**Figure 5:**
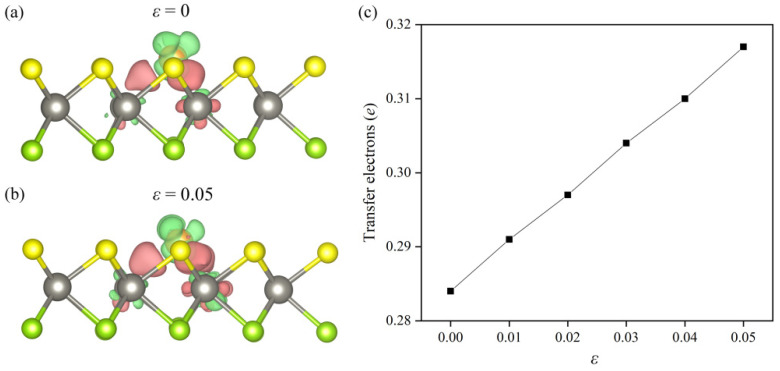
The 3D differential charge density plots of Au/WSSe (**a**) without and (**b**) with external tensile strain. The green and pink regions stand for electron depletion and accumulation, where the isosurfaces are set to 0.004 e/Å^3^. (**c**) The charge gained by the single Au atom in Au/WSSe under different tensile strain.

Generally, orbital hybridization arises with electron transfer. Therefore, in the following work, we studied the electron gained by the single Au atom in the Au/WSSe on the basis of charge density difference (Δ*ρ*) and Bader charge analysis. The Δ*ρ* is defined as follows [42],

$$\Delta \rho =\rho_{\mathrm{Au}/\mathrm{WSSe}}-\rho_{\mathrm{Au}}-\rho_{\mathrm{vac}-s}$$

where $\rho_{\mathrm{Au}/\mathrm{WSSe}}$, $\rho_{\mathrm{Au}}$, and $\rho_{\mathrm{vac}-s}$ are the charge densities of Au/WSSe, single Au atom, and the WSSe monolayer with one S vacancy, respectively. From the electron redistribution shown in Figure 5a, it could be easily seen that there was some electron accumulation (pink area) around the single Au atom. The Bader charge analysis demonstrated that the single Au atom received 0.284 *e* from the support. The electron transfer process was able to separately make the single Au atom and the support become negatively and positively charged. Then, the binding strength between them was reinforced by the electrostatic force originated from the opposite polar components. For the case of Au/WSSe under extra tensile strain (ε = 5%), as illustrated in Figure 5b, the electron accumulation (pink area) around the single Au atom became larger, and more electrons migrated to the single Au atom from the substrate (see Figure 5c), which strengthened the bonding between the single Au atom and the substrate by raising the opposite polarization. Therefore, the Au/WSSe became more stable under larger tensile strain, in line with the results of formation energy shown in Figure 2.

### 3.4. Tunable Electric-Catalytic Performance of Au/WSSe for HER with Tensile Strain

**Figure 6:**
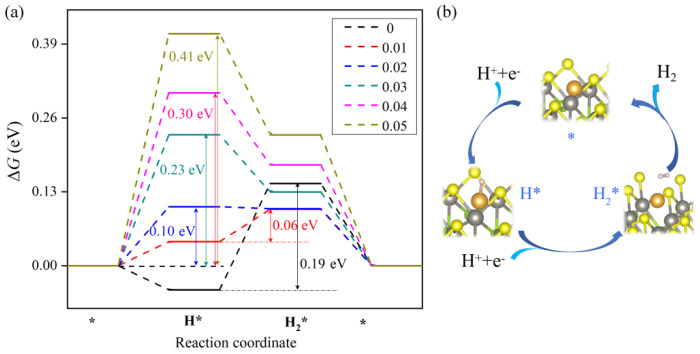
(**a**) The free-energy profile for electrochemical HER on Au/WSSe under different tensile strain. (**b**) The optimized configurations of the intermediates in the HER process. * stands for the adsorption site.

Due to the high hydrogen adsorption free energy ($\Delta G_{H*}=1.82\;\mathrm{eV}$) [43], the pristine Janus WSSe monolayer is inert to the electric-catalytic HER, which is similar to other layered transition metal dichalcogenides [44,45]. Nevertheless, for the Au/WSSe, as shown in Figure 6a, the Δ*G*_H*_ dropped down to −0.042 eV, due to the high activity of the single Au atom. Unfortunately, the second hydrogenation step (H* → H_2_*, see Figure 6b) proceeded a bit laboriously ($\Delta G_{H_{2}*}=0.19\;\mathrm{eV}$), because of the over-powerful binding strength of the intermediate H*. Many previous works reported that the electric-catalytic performance of SAC was sensitive to the electron distribution of the single metal atom [1,3,4]. In addition, as stated earlier, the gained electron of the single Au atom in Au/WSSe could be adjusted by the extra tensile strain. Hereby, we calculated the $\Delta G_{H*}$ on Au/WSSe under different additional tensile strain. As shown in Figure 6a, the $\Delta G_{H*}$ rises as the extra tensile strain becomes heavier.

**Figure 7:**
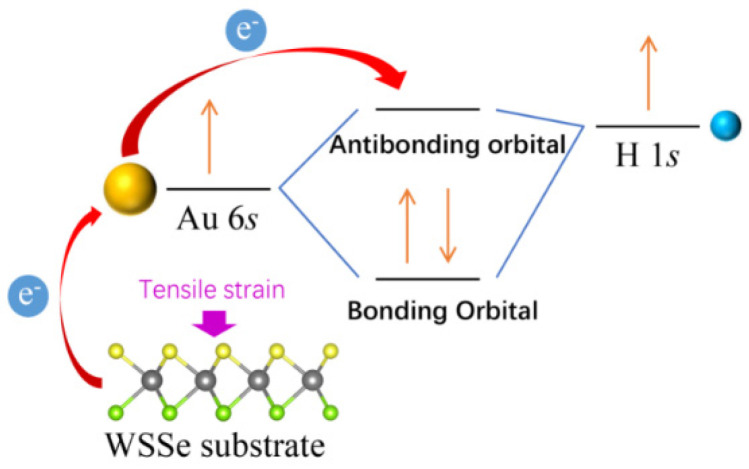
The schematic diagram of the mechanism of external tensile strain affecting adsorption capacity of Au/WSSe for H atom.

One possible explanation for this phenomena mentioned above could be as illustrated in Figure 7. The intrinsic 6*s* electron of the single Au atom combines with the 1*s* electron of the H atom to form the bonding orbital, while the gained electron of the single Au atom from the support has to fill the empty antibonding orbital. As the external tensile strain increases, the antibonding orbital is enhanced with more gained electrons of the single Au atom filled, making the adsorption capacity of Au/WSSe for H atom decline. Furthermore, in view of the whole HER, the reaction barriers on Au/WSSe under relative smaller tensile strain (ε = 1% and 2%) are lower than those on the free Au/WSSe. Especially, in the case of ε = 1%, the reaction barrier (0.06 eV) is only one third of the corresponding one on free Au/WSSe, indicating the application of appropriate tensile strain could improve the electric-catalytic performance of Au/WSSe.

## 4. Conclusions

SACs optimize the usage of metal atoms, which is crucial for supported noble metal catalysts, in particular. Furthermore, SACs have considerable promise for obtaining high activity and selectivity thanks to their homogeneous and well-defined single-atom dispersion. In this work, on the basis of energetic stability, we found that a single Au atom that adsorbs on the surface of perfect WSSe monolayer tends to aggregate; meanwhile, filling the single Au atom at the site of the S vacancy in the WSSe monolayer to build Au/WSSe could keep the single Au atom dispersed, predicting a potential path for fabricating SAC. The powerful binding between the single Au atom and the support in Au/WSSe is induced by the Au *d* and W *d* orbital hybridization, which is caused by the electron transfer between them. The extra tensile strain could further stabilize the Au/WSSe by raising the transfer electron and enhancing the orbital hybridization. Moreover, the extra tensile strain also is able to tune the electric-catalytic HER performance of Au/WSSe by changing the antibonding strength between the single Au atom and H atom. According to the Sebastian principles, the suitable application of extra tensile strain could accelerate the HER rate by reducing the reaction barriers. Especially, with the application of 1% tensile strain, the reaction barrier of HER is merely 0.06 eV, which is smaller than most common electrocatalysts. To sum up, for the first time, our work not only reveals the coupling between the atomic sites and supports in the SAC of Au/WSSe, but also proposes an effective path to improve its electric-catalytic HER performance by tuning the coupling with appropriate tensile strain.

## Data Availability

The data presented in this study are available in Supplementary Materials.

## Supplementary Materials

The following supporting information can be downloaded at: https://www.mdpi.com/article/10.3390/nano12162793/s1, Figure S1: The blue dashed circle indicates the adsorption sites considered in our work at the S and Se layers; Figure S2: The *ab initio* molecular dynamics (AIMD) simulations of Au/WSSe for 5 ps with a time step of 1 fs at 300 K; Figure S3: The optimized configurations of Cu/WSSe and Ag/WSSe, as well as the formation energy (*E*_for_) for each system; Figure S4: Atomic configurations for the diffusion of the Au single atoms from one favourable doping site to its neighbouring one at the defective WSSe surface at free state and under 5% tensile strain, including initial state, transition state and final state; Figure S5: Calculated projected density of states at PBE level for pristine WSSe monolayer, single Au atom adsorbed WSSe monolayer, and free Au/WSSe; Figure S6: The band structure of Au/WSSe obtained with HSE06 functional; Table S1: Zero-pint Energy Correction, Entropy Contribution, Total Energy, and the Gibbs Free Energy of H^*^ and H_2_^*^Adsorbates on Au/WSSe under different tensile strain; Detailed calculation information on the free energy differences of hydrogenation steps for HER [24].

## References

[1] Ju L., Tan X., Mao X., Gu Y., Smith S., Du A., Chen Z., Chen C., Kou L. (2021). Controllable CO_2_ electrocatalytic reduction via ferroelectric switching on single atom anchored In_2_Se_3_ monolayer. Nat. Commun..

[2] Hannagan R.T., Giannakakis G., Flytzani-Stephanopoulos M., Sykes E.C.H. (2020). Single-Atom Alloy Catalysis. Chem. Rev..

[3] Zhang Y., Yang J., Ge R., Zhang J., Cairney J.M., Li Y., Zhu M., Li S., Li W. (2022). The effect of coordination environment on the activity and selectivity of single-atom catalysts. Coordin. Chem. Rev..

[4] Xu H., Zhao Y., Wang Q., He G., Chen H. (2022). Supports promote single-atom catalysts toward advanced electrocatalysis. Coordin. Chem. Rev..

[5] Li Z., Ji S., Liu Y., Cao X., Tian S., Chen Y., Niu Z., Li Y. (2020). Well-Defined Materials for Heterogeneous Catalysis: From Nanoparticles to Isolated Single-Atom Sites. Chem. Rev..

[6] Wang A., Li J., Zhang T. (2018). Heterogeneous single-atom catalysis. Nat. Rev. Chem..

[7] Su J., Ge R., Dong Y., Hao F., Chen L. (2018). Recent progress in single-atom electrocatalysts: Concept, synthesis, and applications in clean energy conversion. J. Mater. Chem. A.

[8] Quan Z., Wang Y., Fang J. (2013). High-Index Faceted Noble Metal Nanocrystals. Accounts Chem. Res..

[9] Lu A.Y., Zhu H., Xiao J., Chuu C.P., Han Y., Chiu M.H., Cheng C.C., Yang C.W., Wei K.H., Yang Y. (2017). Janus monolayers of transition metal dichalcogenides. Nat. Nanotechnol..

[10] Ju L., Bie M., Zhang X., Chen X., Kou L. (2021). Two-dimensional Janus van der Waals heterojunctions: A review of recent research progresses. Front. Phys..

[11] Ju L., Bie M., Shang J., Tang X., Kou L. (2020). Janus transition metal dichalcogenides: A superior platform for photocatalytic water splitting. J. Phys. Mater..

[12] Ju L., Bie M., Tang X., Shang J., Kou L. (2020). Janus WSSe Monolayer: An Excellent Photocatalyst for Overall Water Splitting. ACS Appl. Mater. Interfaces.

[13] Ma X., Wu X., Wang H., Wang Y. (2018). A Janus MoSSe monolayer: A potential wide solar-spectrum water-splitting photocatalyst with a low carrier recombination rate. J. Mater. Chem. A.

[14] Ma X., Yong X., Jian C.-C., Zhang J. (2019). Transition Metal-Functionalized Janus MoSSe Monolayer: A Magnetic and Efficient Single-Atom Photocatalyst for Water-Splitting Applications. J. Phys. Chem. C.

[15] Tao S., Xu B., Shi J., Zhong S., Lei X., Liu G., Wu M. (2019). Tunable Dipole Moment in Janus Single-Layer MoSSe via Transition-Metal Atom Adsorption. J. Phys. Chem. C.

[16] Xiong Y., Dong J., Huang Z.Q., Xin P., Chen W., Wang Y., Li Z., Jin Z., Xing W., Zhuang Z. (2020). Single-atom Rh/N-doped carbon electrocatalyst for formic acid oxidation. Nat. Nanotechnol..

[17] Chen Y., Ji S., Chen C., Peng Q., Wang D., Li Y. (2018). Single-Atom Catalysts: Synthetic Strategies and Electrochemical Applications. Joule.

[18] Kresse G., Furthmüller J. (1996). Efficient iterative schemes for ab initio total-energy calculations using a plane-wave basis set. Phys. Rev. B.

[19] Kresse G., Furthmüller J. (1996). Efficiency of ab-initio total energy calculations for metals and semiconductors using a plane-wave basis set. Comput. Mater. Sci..

[20] Blöchl P.E. (1994). Projector augmented-wave method. Phys. Rev. B.

[21] Kresse G., Joubert D. (1999). From ultrasoft pseudopotentials to the projector augmented-wave method. Phys. Rev. B.

[22] Perdew J.P., Burke K., Ernzerhof M. (1996). Generalized Gradient Approximation Made Simple. Phys. Rev. Lett..

[23] Grimme S. (2006). Semiempirical GGA-type density functional constructed with a long-range dispersion correction. J. Comput. Chem..

[24] Nørskov J.K., Rossmeisl J., Logadottir A., Lindqvist L., Kitchin J.R., Bligaard T., Jónsson H. (2004). Origin of the Overpotential for Oxygen Reduction at a Fuel-Cell Cathode. J. Phys. Chem. B.

[25] Ju L., Shang J., Tang X., Kou L. (2020). Tunable Photocatalytic Water Splitting by the Ferroelectric Switch in a 2D AgBiP_2_Se_6_ Monolayer. J. Am. Chem. Soc..

[26] Mao X., Kour G., Zhang L., He T., Wang S., Yan C., Zhu Z., Du A. (2019). Silicon-doped graphene edges: An efficient metal-free catalyst for the reduction of CO_2_ into methanol and ethanol. Catal. Sci. Technol..

[27] Liu H., Cheng J., He W., Li Y., Mao J., Zheng X., Chen C., Cui C., Hao Q. (2022). Interfacial electronic modulation of Ni_3_S_2_ nanosheet arrays decorated with Au nanoparticles boosts overall water splitting. Appl. Catal. B-Environ..

[28] Guo M., Du J. (2012). First-principles study of electronic structures and optical properties of Cu, Ag, and Au-doped anatase TiO_2_. Phys. B.

[29] Yang Z., Wu R., Goodman D.W. (2000). Structural and electronic properties of Au on TiO_2_ (110). Phys. Rev. B.

[30] Zada A., Humayun M., Raziq F., Zhang X., Qu Y., Bai L., Qin C., Jing L., Fu H. (2016). Exceptional Visible-Light-Driven Cocatalyst-Free Photocatalytic Activity of g-C_3_N_4_ by Well Designed Nanocomposites with Plasmonic Au and SnO_2_. Adv. Energy Mater..

[31] Humayun M., Ullah H., Cao J., Pi W., Yuan Y., Ali S., Tahir A.A., Yue P., Khan A., Zheng Z. (2019). Experimental and DFT Studies of Au Deposition Over WO_3_/g-C_3_N_4_ Z-Scheme Heterojunction. Nanomicro Lett..

[32] Ghosh D.C., Chakraborty T. (2009). Gordy’s electrostatic scale of electronegativity revisited. J. Mol. Struc. THEOCHEM.

[33] Ma D., Ju W., Li T., Zhang X., He C., Ma B., Lu Z., Yang Z. (2016). The adsorption of CO and NO on the MoS_2_ monolayer doped with Au, Pt, Pd, or Ni: A first-principles study. Appl. Surf. Sci..

[34] Ju L., Liu P., Yang Y., Shi L., Yang G., Sun L. (2021). Tuning the photocatalytic water-splitting performance with the adjustment of diameter in an armchair WSSe nanotube. J. Energy Chem..

[35] Liu D., Li X., Chen S., Yan H., Wang C., Wu C., Haleem Y.A., Duan S., Lu J., Ge B. (2019). Atomically dispersed platinum supported on curved carbon supports for efficient electrocatalytic hydrogen evolution. Nat. Energy.

[36] Jin C., Tang X., Tan X., Smith S.C., Dai Y., Kou L. (2019). A Janus MoSSe monolayer: A superior and strain-sensitive gas sensing material. J. Mater. Chem. A.

[37] Liu S., Yin H., Singh D.J., Liu P.-F. (2022). Ta_4_SiTe_4_: A possible one-dimensional topological insulator. Phys. Rev. B.

[38] Heyd J., Scuseria G.E., Ernzerhof M. (2003). Hybrid functionals based on a screened Coulomb potential. J. Chem. Phys..

[39] Wan G., Lin X.-M., Wen J., Zhao W., Pan L., Tian J., Li T., Chen H., Shi J. (2018). Tuning the Performance of Single-Atom Electrocatalysts: Support-Induced Structural Reconstruction. Chem. Mater..

[40] Yuan S., Pu Z., Zhou H., Yu J., Amiinu I.S., Zhu J., Liang Q., Yang J., He D., Hu Z. (2019). A universal synthesis strategy for single atom dispersed cobalt/metal clusters heterostructure boosting hydrogen evolution catalysis at all pH values. Nano Energy.

[41] O’Connor N.J., Jonayat A.S.M., Janik M.J., Senftle T.P. (2018). Interaction trends between single metal atoms and oxide supports identified with density functional theory and statistical learning. Nat. Catal..

[42] Ju L., Dai Y., Wei W., Li M., Huang B. (2018). DFT investigation on two-dimensional GeS/WS_2_ van der Waals heterostructure for direct Z-scheme photocatalytic overall water splitting. Appl. Surf. Sci..

[43] Er D., Ye H., Frey N.C., Kumar H., Lou J., Shenoy V.B. (2018). Prediction of Enhanced Catalytic Activity for Hydrogen Evolution Reaction in Janus Transition Metal Dichalcogenides. Nano Lett..

[44] Zhang J., Jia S., Kholmanov I., Dong L., Er D., Chen W., Guo H., Jin Z., Shenoy V.B., Shi L. (2017). Janus Monolayer Transition-Metal Dichalcogenides. ACS Nano.

[45] Li H., Tsai C., Koh A.L., Cai L., Contryman A.W., Fragapane A.H., Zhao J., Han H.S., Manoharan H.C., Abild-Pedersen F. (2016). Corrigendum: Activating and optimizing MoS_2_ basal planes for hydrogen evolution through the formation of strained sulphur vacancies. Nat. Mater..

