# Supplementary material for: Improving the Energetic Stability and Electrocatalytic Performance of Au/WSSe Single-Atom Catalyst with Tensile Strain
Source: Nanomaterials (Basel). 2022 Aug 15;12(16):2793. doi: 10.3390/nano12162793 (PMC9414615; doi:10.3390/nano12162793)
Supplement: Supplementary file 1 [file nanomaterials-12-02793-s001.zip › nanomaterials-1816629-supplementary.pdf]

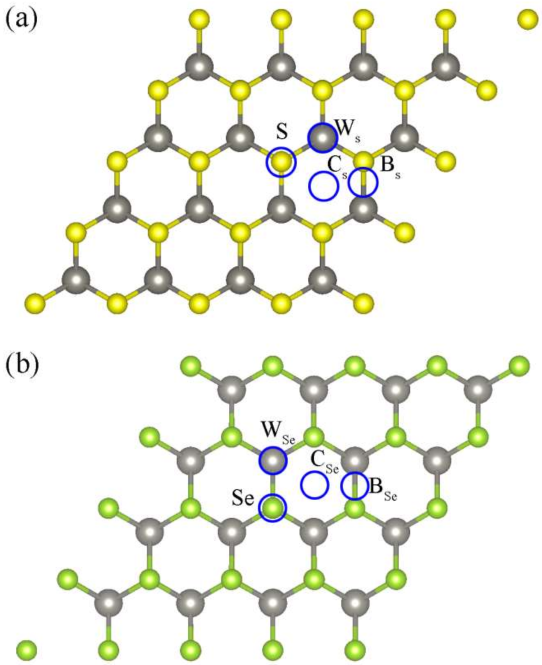

Figure S1. The blue dashed circle indicates the adsorption sites considered in our work at the (a) S and (b) Se layers. The yellow, green and gray balls represent the S, Se, and W atoms, respectively.

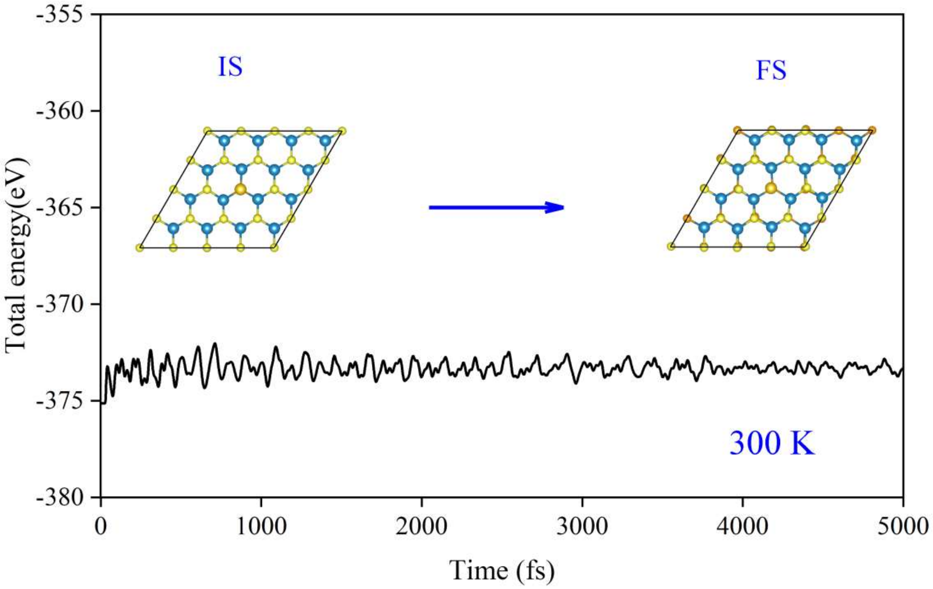

Figure S2. The *ab initio* molecular dynamics (AIMD) simulations of Au/WSSe for 5 ps with a time step of 1 fs at 300 K. The insert shows the morphologic structures of initio state (IS) and final state (FS).

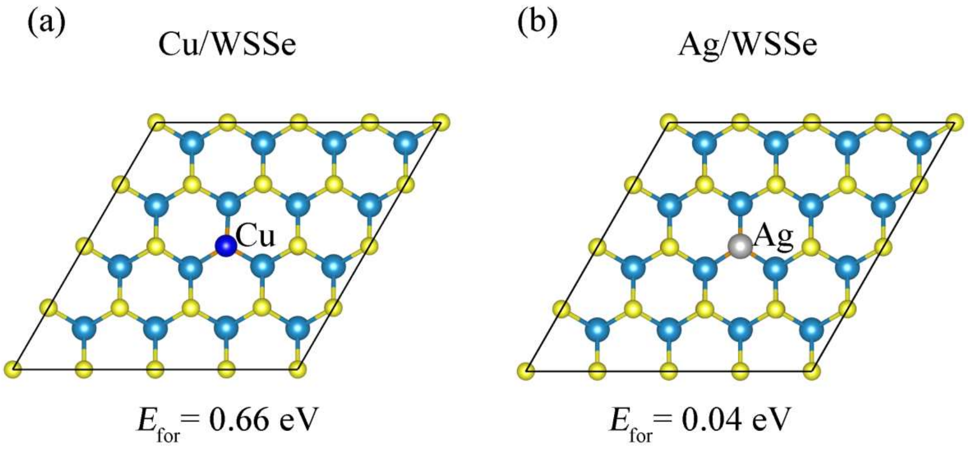

Figure S3. The optimized configurations of Cu/WSSe (a) and Ag/WSSe (b), as well as the formation energy (E_{for}) for each system.

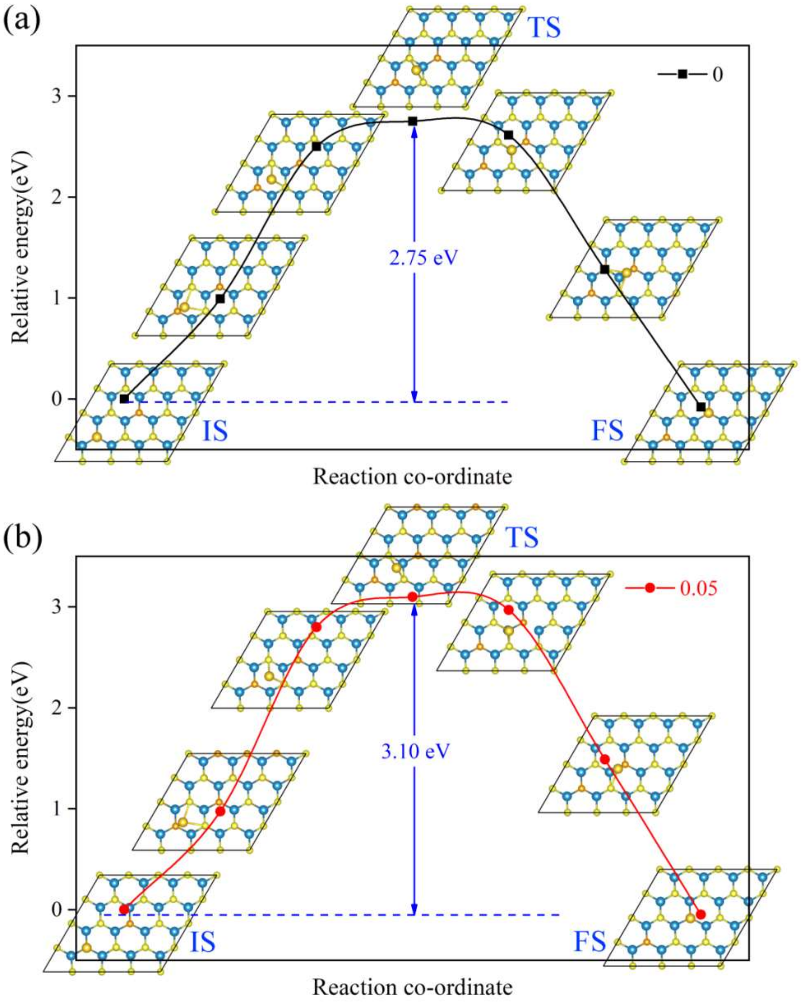

Figure S4. Atomic configurations for the diffusion of the Au single atoms from one favourable doping site to its neighbouring one at the defective WSSe surface (a) at free state and (b) under 5% tensile strain, including initial state (IS), transition state (TS) and final state (FS). The energy is given with respect to IS.

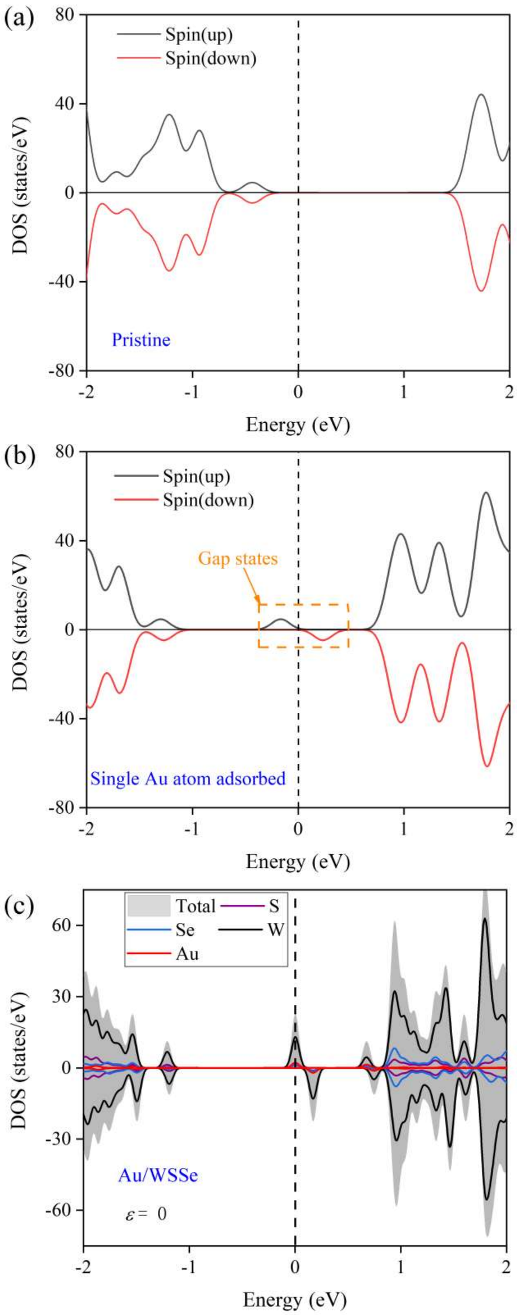

Figure S5. Calculated projected density of states at PBE level for (a) pristine WSSe monolayer, (b) single Au atom adsorbed WSSe monolayer, and (c) free Au/WSSe. The Fermi level as indicated by the dashed line is set as 0 eV.

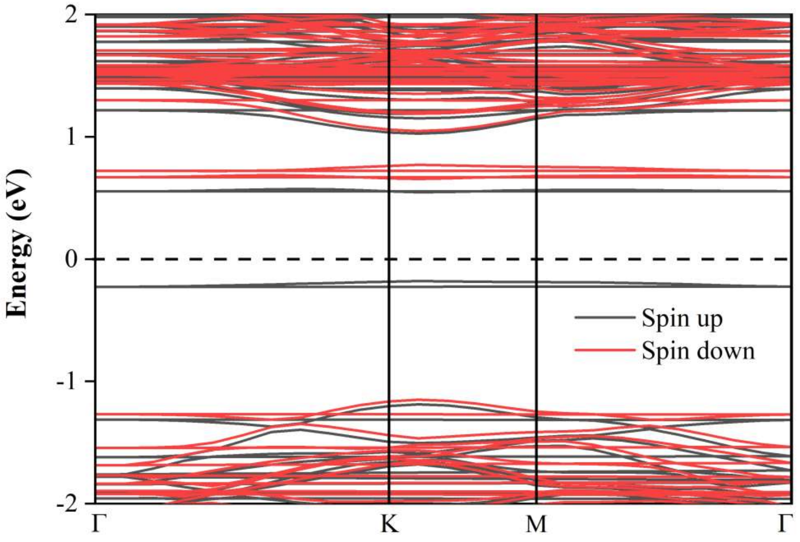

Figure S6. The band structure of Au/WSSe obtained with HSE06 functional. The Fermi level (the dashed line) is set to 0 eV.

Free energy difference (ΔG)

Free energy difference (ΔG) in the hydrogen evolution reactions is calculated according the approach proposed by Nørskovet *al.* [1] The formula at pH = 0 without solar irradiation can be defined as below:

$$\Delta G = \Delta E + \Delta E_{zpe} - T\Delta S \qquad \text{(S1)}$$

where ΔE is the formation energy, ΔE_{zpe} and ΔS are the difference in zero point energy and entropy difference between the adsorbed state and the gas phase, respectively.

Meanwhile, hydrogen production half reaction can be decomposed into two steps, the reaction equation can be written as:

$$* + H^{+} + e^{-} \rightarrow H* \qquad \text{(a)}$$

$$*H + H^{+} + e^{-} \rightarrow H_2* \qquad \text{(b)}$$

$$H_2* \rightarrow * + H_2 \qquad \text{(b)}$$

where * means the adsorbed materials, while H* and H_2* represent the adsorbed intermediates.

For each reaction of hydrogen production, the free energy difference under the effect of pH and an extra potential bias can be written as:

$$\Delta G_a = G_{H*} - \frac{1}{2}G_{H_2} - G_* + \Delta G_U + \Delta G_{pH} \qquad \text{(S2)}$$

$$\Delta G_b = G_{H_2*} - \frac{1}{2}G_{H_2} - G_{H*} + \Delta G_U + \Delta G_{pH} \qquad \text{(S3)}$$

$$\Delta G_c = G_* + G_{H_2} - G_{H_2*} + \Delta G_U + \Delta G_{pH} \qquad \text{(S4)}$$

Where ΔG_{pH} ($\Delta G_{pH} = k_B T \times ln10 \times pH$) represents the free energy contributed in different pH concentration. ΔG_U ($\Delta G_U = -eU$) denotes extra potential bias provided by an electron in the electrode, where U is the electrode potential relative to the standard hydrogen electrode (SHE).

Table S1 Zero-pint Energy Correction (E_{ZPE}), Entropy Contribution (TS, T=298.15 K), Total Energy (E), and the Gibbs Free Energy (G) of H^* and H_2^*Adsorbates on Au/WSSe under different tensile strain.

species	Tensile	E_{ZPE}(eV)	-TS (eV)	E (eV)	G (eV)
H^*	0.00	0.141	-0.0568	-378.633	-378.548
	0.01	0.149	-0.0440	-378.536	-378.431
	0.02	0.121	-0.0527	-378.303	-378.235
	0.03	0.117	-2.956×10^{-5}	-377.98	-377.864
	0.04	0.116	-3.007×10^{-5}	-377.557	-377.441
	0.05	0.116	-3.034×10^{-5}	-377.04	-376.924
H_2^*	0.00	0.330	-0.131	-382.015	-381.816
	0.01	0.291	-0.173	-381.946	-381.829
	0.02	0.288	-0.174	-381.807	-381.693
	0.03	0.297	-0.159	-381.558	-381.420
	0.04	0.309	-0.125	-381.206	-381.023
	0.05	0.314	-0.114	-380.757	-380.557

1. Nørskov, J. K.; Rossmeisl, J.; Logadottir, A.; Lindqvist, L.; Kitchin, J. R.; Bligaard, T.; Jónsson, H., Origin of the Overpotential for Oxygen Reduction at a Fuel-Cell Cathode. *J. Phys. Chem. B* **2004,** 108, (46), 17886-17892.
